# An Overview of Nutritional Aspects in Juvenile Idiopathic Arthritis

**DOI:** 10.3390/nu14204412

**Published:** 2022-10-20

**Authors:** Renata Puppin Zandonadi

**Affiliations:** Department of Nutrition, Faculty of Health Sciences, Campus Universitário Darcy Ribeiro, University of Brasília, Brasilia 70910-900, Brazil; renatapz@unb.br; Tel.: +55-61-981033600

**Keywords:** juvenile idiopathic arthritis, dietary supplements, nutrient, diet

## Abstract

There is evidence that nutritional impairment can complicate juvenile idiopathic arthritis (JIA). It is also recognized that the JIA drug treatment may affect the nutritional aspects of patients. It is crucial to understand the impacts that nutritional aspects can have on a patient’s treatment, health, and life. Therefore, this review explores how nutrition influences juvenile idiopathic arthritis. Dietary aspects play essential roles in JIA patients’ growth, body mass index (BMI), bone mineral density (BMD), inflammation, and recovery. Suboptimal nutrition seems to adversely affect the long-term outcome of JIA patients. Nutritional deficiency potentially affects JIA patients’ general wellbeing and disease control and contributes to growth, inflammation, BMI, and BMD disturbances. It was also possible to verify that the correct status of nutrients helps the body recover and reduce inflammation in JIA patients, since nutritional status and nutrients play an important role in regulating immune function. Studies are diverse, and most analyze the effects of a single nutrient on JIA. Moreover, the diet and nutrition impacts are difficult to interpret in the pediatric population due to family influence, dietary regulation, and data collection in children/adolescents. Despite the lack of standardization among studies, the potential benefits of a healthy diet on short- and long-term health and wellbeing in JIA patients are noteworthy.

## 1. Introduction

Juvenile idiopathic arthritis (JIA) is the most common type of arthritis in children and adolescents and can cause joint damage, chronic pain, and disability [1,2]. It is characterized as a group of different forms of arthritis of unknown origin occurring in children and adolescents starting before 16 years old and persisting for more than six weeks [1,3,4]. JIA affects more than 2 million patients worldwide, with a prevalence of 16–50 cases per 100,000 individuals, varying in ethnicity [2,5]. Since JIA has been increasing worldwide, its management has become highlighted in pediatric care [6].

JIA types are autoimmune or autoinflammatory diseases and can typically cause inflammation and/or pain in one or more joints, mainly in the hands, knees, ankles, elbows, and wrists, but may also affect other body parts [1]. JIA disorders have been grouped based on clinical and laboratory features to identify homogeneous, mutually exclusive categories. These features allow the characterization of some JIA categories, but some still include heterogeneous disorders that are not well characterized [3,7].

JIA and its drug treatment may affect the nutritional aspects of patients, resulting in nutritional impairment [6,8,9,10,11]. Therefore, it is crucial to understand the impacts that JIA can have on the patient’s treatment, health, and life. Despite nutritional impairment being recognized as a JIA complication, factors associated with it are not broadly studied. Suboptimal nutrition potentially affects the long-term outcome of JIA patients and concerns them and their parents [12]. Nutritional impairment affects the JIA patients’ general wellbeing, and may affect growth and disease control [11]. A healthy diet is essential for children and adolescents, but for those with JIA, it represents an additional challenge: eating foods that promote adequate growth and development but also help reduce inflammation and support their health and immune system, since diet plays a crucial role in the inflammatory processes [12]. Despite JIA treatment being based on medicines, biological treatments, and other interventions, exploring dietary changes may support better management of JIA [13,14]. Therefore, this review explores how nutrition influences juvenile idiopathic arthritis.

## 2. Body Composition and Dietary Challenges in JIA

JIA patients tend to present body composition and growth impairment due to disease activity, medication, reduced physical activity, and/or malnutrition [1,8,15]. Growth impairment is common among JIA patients, ranging from retarded growth to local acceleration of growth in the body part affected, associated with increased production of pro-inflammatory cytokines (interleukin-1β-IL-1β, tumor necrosis factor-α-TNF-α, and interleukin-6-IL-6) [16]. A study evaluated linear growth and final height in JIA patients (n = 24) given glucocorticoids during childhood [17]. During glucocorticoid therapy, mean loss of chronological age was positively correlated with therapy duration. After glucocorticoid discontinuation, 70% of JIA patients had catch-up growth. Mean final height was significantly greater in the patients with catch-up growth at glucocorticoid discontinuation, but 87% of patients had a final height below their target height, suggesting that chronic inflammation and prednisone therapy may adversely affect growth in JIA patients [17]. One hypothesis for short stature in JIA patients is inadequate dietary intake. A study assessed the nutritional status of 34 JIA patients and nine healthy controls by 3-day diet records, anthropometries, and biochemical analyses [18]. One-third of JIA patients were at or below the 10th percentile in height for age. However, the mean dietary intake for calories and essential nutrients was found to be adequate. No significant correlations were found between dietary intake and growth percentiles in the studied groups [18]. Biochemical abnormalities were found among JIA patients, including low plasma levels of vitamins A and C, proteins, and zinc, and increased levels of copper and glutathione peroxidase activity [18]. No changes in plasma selenium and vitamin E levels were observed. The authors suggested that the divergence between intake and low levels of circulating nutrients may come from alterations in the requirements, absorption, or use of these nutrients in JIA chronic inflammation [18].

A cohort study followed 1147 Canadian JIA patients for about 35.5 months and showed that about 10% of JIA patients with uncontrolled disease and/or prolonged corticosteroid use had an increased risk of growth impairment. However, the authors concluded that most JIA patients analyzed grew and gained weight similarly to North American populations [19]. Another cohort study evaluated the three-year vertical growth patterns and factors associated with poor growth in 568 JIA patients in the U.K. [20]. The authors showed that 39% of JIA patients experienced an important loss in vertical growth over the first three years of the disease. Moreover, 23% (n = 118) of them were overweight or obese, probably due to poor growth in height in these patients [20]. Another cohort study evaluated 30 Indian JIA patients for five years and showed that they had decreased height and growth retardation [21].

A study reported significant growth restriction in JIA patients with no exposure to corticosteroids, suggesting that growth restriction in JIA is complex and may be related directly to the inflammatory process, since high levels of IL-6 may reduce growth velocity [22]. It is evident that a healthy diet contributes to growth; however, in JIA patients, factors related to inflammation and medication seem to be the primary causes of growth impairment [17,19,20,22,23]. In JIA patients, several nutrients can effectively reduce inflammation, potentially benefitting growth indirectly. However, no study was found that searches for the association between diet and growth in JIA patients.

JIA patients tend to present a decreased lean mass and an increased fat mass and present higher energy requirements because of the higher protein catabolism induced by cytokines compared to healthy individuals [23,24,25]. However, calorie intake may be impaired by appetite loss due to anorexigenic cytokines’ effect, pain, sleeping, or depressive disturbances. These factors may lead to an imbalance between the number of calories consumed and calorie requirements due to JIA. It is estimated that protein/energy deficiency occurs in 20–40% of JIA patients [10,23,26]. A study evaluated the body composition in 46 JIA patients compared to healthy children and showed that JIA patients were at a higher risk of malnutrition than their healthy peers, predominantly in patients with polyarthritis, but the dietary aspects were not evaluated [27]. Another study assessed the nutrient intake and nutritional status of 15 JIA patients compared to 17 healthy controls [28]. Concentrations of hemoglobin, serum iron, and zinc were lower and serum copper was higher in JIA patients than control. JIA patients presented impaired nutrition despite the increased energy and protein intake compared to control, indicating that the inflammatory process leads to increased energy metabolism [28]. 

Differently, some studies indicate a higher prevalence of overweight and obesity in JIA patients [8,19,29]. A study investigated the dietary intake of Brazilian JIA patients (n = 48) using a 24-hour diet recall and relating it to the patients’ characteristics and the medications used in their treatment [9]. The results showed that JIA patients were mostly of a normal weight (n = 26; 54%) and 17% (n = 8) were obese. Most JIA participants presented low consumption of micronutrients, mainly vitamin E (89%), vitamin A (87%), zinc (87%), and calcium (62%). The JIA patients did not show a significant association between energy intake, macronutrients, and micronutrients and BMI, disease activity, use of methotrexate, oral corticoid, or methylprednisolone. The results revealed low consumption of dairy products, fruits, and vegetables and high consumption of lipids, sugar, and sweets. More than 30% of JIA participants consumed fried food, soft drinks, artificial juice, and/or coffee [9]. However, the authors mentioned that their results were similar to those found in healthy Brazilian children and adolescents [9], emphasizing the importance of nutritional education to improve healthy eating habits at this age, mainly for JIA patients with an inflammatory condition.

Another study compared JIA patients’ and healthy peers’ body composition, energy intake, and nutritional biomarkers [30]. The results showed that JIA patients with low disease activity have adiposity and a higher energy intake than controls. About 30% of the JIA patients and 12.5% of the healthy controls were overweight or obese. Neither disease subtype nor disease activity were associated with changes in body composition [30]. The authors showed that early JIA patients had low BMI, while JIA patients with a longer disease duration had a BMI higher than or similar to healthy controls [30]. However, they pointed out as a limitation that they did not analyze the effect of medication on nutritional parameters and body composition [30]. 

A cross-sectional study conducted with Moroccan JIA patients (n = 33) evaluated the relationship between macronutrient intake (using 7-day 24-hour recall), body composition, and bone mineral content (using DXA total body measurements) [15]. The study showed a positive correlation between carbohydrate intake and lean body mass, but dietary intake did not influence fat mass and bone mineral content [15].

In JIA patients, there are several causes of growth delay, such as increased catabolic demands secondary to active disease, decreased levels of insulin-like growth factor 1 (ILGF-1) in children secondary to elevated levels of IL-6 and corticosteroids, suppressive effect of corticoids on osteoblasts, and those related to malnutrition such as cachexia associated with increased levels of TNF-a and IL-1 and anorexia related to medication side effects [31].

Another important point was revealed by a recent multinational, cross-sectional, observational cohort study that evaluated 9081 JIA patients from 49 countries. The study reported worse clinical outcomes in patients from countries with lower gross domestic product (GDP) [32]. However, the study did not evaluate aspects related to food access and diet and only mentioned that the disparities could partly be related to unequal access to the costly biological disease-modifying antirheumatic drugs and the delay in access to rheumatology care [32]. Despite the absence of studies on food security for JIA patients, food insecurity in lower GDP countries could also be related to worse JIA outcomes in low-income patients.

### Special Dietary Patterns

A study explored the prevalence of special diet adoption in JIA patients and parental perceptions of efficacy with 261 parents of JIA patients in the United States [13]. About 30% of participants (n = 79) followed special diets (gluten-free—66%; anti-inflammatory—41%; lactose-free—25%; vegetarian/vegan—20%). About 50% of parents whose children adopted special diets perceived improvement in pain or joint swelling. When looking at medication changes while following a special diet, in the patient group adopting a gluten-free diet, 19% escalated medication, 8% reduced its use, and 73% reported no change [13]. A different trend occurred with the lactose-free diet: 20% of patients reduced the use of antirheumatic medication, 70% reported no change, and none had an escalation in antirheumatic medication. The responses from other dietary patterns were not mentioned [13]. Due to the nature of the study that evaluated parental perception and the limited number of participants adopting special diets, it is not possible to affirm if the special diets improved pain or reduced the use of medication or if this is the result of the therapies adopted concomitantly with the change in the dietary pattern. However, it is important to consider adopting different dietary patterns in further studies with JIA patients since there is evidence of improvement in rheumatoid arthritis in general when adopting different dietary patterns, such as vegetarian and gluten-free diets [33,34].

A pilot study with four JIA patients in Finland evaluated the 6-month gluten-free diet (GFD) attenuating joint symptoms in these patients [35]. GDF showed a modest improvement in articular symptoms in two non-celiac JIA children. However, the authors mentioned that the results were inconclusive, needing a more robust study including more active non-celiac JIA patients [35]. 

A study conducted in a Swedish showed that fish consumption more than once per week during pregnancy or in the first year of a child’s life was associated with an increased risk of JIA, probably due to the exposure to heavy metals related to fish consumption during pregnancy or early childhood [14]. The authors found higher levels of aluminum, mercury, cadmium, and lithium in the cord blood of children with JIA than in controls, supporting the hypothesis that the association is mediated by exposure to heavy metals in fish [14]. 

Recently, a pilot study investigated if the improved clinical symptoms after exclusive enteral nutrition (EEN) and a specific carbohydrate diet (SCD) were linked to changes in the fecal microbiota of 16 Swedish JIA patients (6 EEN—3 to 5 weeks; 10 SCD—4–5 weeks) [36]. SCD is a diet without many complex carbohydrates such as grains, dairy products (except fermented yogurt), vegetables rich in starch, and sugars (except monosaccharides, such as those in honey). Analyses of the fecal microbiota showed an effect on the overall composition of these special diets. Fecal samples from before and during treatment showed an impact on the fecal microbiota composition and a numerical reduction in α-diversity for both dietary interventions, with a significant decrease in SCD [36]. The authors suggested that additional larger studies could clarify the clinical effects of those dietary interventions [36]. 

Another pilot study investigated the anti-inflammatory effects of the specific carbohydrate diet in 15 JIA children completing a 4-week diet [37]. The results showed that SCD decreased children’s morning stiffness and pain and improved physical function. In seven JIA children, inflammatory proteins, including TNF-alpha, decreased. Fecal butyrate from 15 participants increased significantly [37]. The author highlighted that it was challenging to prepare the home-cooked meals required to adopt a special diet, but the improvement in most patients motivates them and their parents. The author speculated that eliminating unhealthy food, additives, and emulsifiers and restricting carbohydrates and dairy products play an immunological role in JIA, but future studies are necessary on the dietary immunological influence in JIA children [37].

According to the American College of Rheumatology, there is little evidence that a specific restrictive diet should be adopted in JIA treatment [38]. Unnecessary adoption of restrictive diets such as gluten- and dairy-free may impair nutritional status and heighten the risk of other harms such as delay in treatment, cost, and inconvenience [38]. No clear evidence supports the adoption of a specific diet alone to treat JIA, but a healthy, well-balanced, age-appropriate diet is needed to support the patient’s health and quality of life [38]. 

## 3. Dietary Aspects in Juvenile Idiopathic Arthritis and Potential Benefits of Nutritional Treatment

### 3.1. Polyunsaturated Fatty Acids (PUFA)

Considering that interleukin-1 (IL-1) and tumor necrosis factor-α (TNF-α) are cytokines that play essential roles in the pathogenesis and prognosis of JIA, a study evaluated the effect of omega-3 fatty acid supplements (2 g/day for 12 weeks) on the clinical manifestations, laboratory investigations, and disease activity in JIA patients [39]. The authors showed that omega-3 fatty acids reduced the inflammatory response and improved the clinical manifestation of JIA. It also decreased the patient daily intake of nonsteroidal anti-inflammatory drugs, suggesting the use of omega-3 fatty acids as an add-on therapy to conventional JIA treatment [39].

Another study aimed to investigate the association between dietary intake of omega-3 and omega-6 PUFA, serum PUFA profile, and immune and inflammatory markers in JIA patients [40]. The authors found higher serum IL-10 levels in JIA patients than in the control group. Omega-6 and omega-3 serum levels were negatively correlated with active joint count and erythrocyte sedimentation rate. Low levels of PUFA were found in the active phase of JIA, showing a potential contribution to the pathogenesis of JIA. The results pointed out a group of JIA patients to whom PUFA supplementation and a regular daily diet might be recommended to have a positive immunomodulating effect [40]. To date, the usefulness of PUFA in JIA treatment is not well established. Further studies are necessary to evaluate the dose response and benefits of incorporating PUFA as an add-on therapy in JIA patients. 

### 3.2. Trans and Saturated Fat

A study evaluated the prevalence of dyslipoproteinemia in 28 Brazilian JIA patients [41]. The authors identified 71% of JIA patients with dyslipoproteinemia, mainly decreased high-density lipoprotein cholesterol (HDLc) (57%), followed by elevated levels of low-density lipoprotein cholesterol (18%), triglycerides (14%), and total cholesterol (7%). Decreased HDLc levels were not related to disease activity, disease duration, or therapy [41]. The authors speculate, although the relationship between physical activity and HDLc levels was not evaluated, that active JIA associated with physical incapacity might affect the dyslipidemic status found in the study [41]. Potentially, diet and drug treatment could also affect the lipid levels observed, but they did not evaluate them. Therefore, it was not possible to establish the leading cause of their findings [41]. Another study with 166 Danish JIA patients [42] showed that JIA patients’ cholesterol levels were in the normal range. However, the level of HDLc was negatively associated with markers of inflammation, following the concept of inflammation as a crucial driver for the early development of atherosclerosis in JIA patients [42]. A cross-sectional study evaluated dyslipidemia in 62 Brazilian JIA patients. Dyslipidemia was frequent in JIA patients (83.3%), although most had good nutritional status (74% normal weight) but inadequate consumption of atherogenic lipids [43]. JIA patients did not present a carbohydrate intake higher than the recommendation, 4.9% had a protein intake higher than the recommendation, and 29% had a lipid intake higher than the recommendation. Almost 76% of JIA patients had saturated fat intake higher than the recommendation, and 79% had a trans-fat intake higher than the recommendation, justifying dyslipidemia in these patients [43]. 

Despite studies showing the association of dyslipidemia with inflammation in JIA patients, the studies have limitations due to their cross-sectional or short-term uncontrolled design. Therefore, a longitudinal study investigated serum lipid levels, atherogenic indices, and their association with the clinical and laboratory parameters of JIA activity in 58 Taiwanese patients [44]. The authors found abnormal lipid levels and atherogenic indices associated with JIA activity. The parameters improved following effective antirheumatic treatment, potentially reducing the risk of cardiovascular disease in JIA [44]. The authors mentioned that despite the correlation between inflammation and lipid profiles in JIA patients, the exact mechanisms remain to be elucidated. Cytokine-induced activation of the reticuloendothelial system is potentially critical to such changes [44]. Considering the findings on JIA and lipid profile, diet could possibly help prevent cardiovascular diseases in JIA patients, but further studies are necessary to elucidate this topic [45].

### 3.3. Fruits and Vegetables

The consumption of fruits and vegetables in JIA patients improves their intake of vitamin C and bioactive compounds that can exert anti-inflammatory and antioxidant activity in their bodies [46]. It is well-known that low consumption of fruits and vegetables can lead to vitamin C deficiency (scurvy), as humans require exogenous vitamin C from foods [47]. Concentrations of antioxidants, including vitamin C, are inversely associated with C-reactive protein and other inflammatory markers. During an inflammatory response, there is an increase in the release of reactive oxygen species in damaged areas, balanced by antioxidants. Therefore, in vitamin C deficiency, it might cause a loss of antioxidant effect, contributing to high inflammatory markers, potentially harming JIA patients [47]. It is important to highlight that fruits and vegetables are part of a healthy diet that can contribute to JIA patients’ health and development and disease management [11,12,18,48].

A study evaluated the efficacy and safety of combination therapy of blueberry and etanercept (an inhibitor of tumor necrosis factor used to treat arthritis rheumatoid) in 201 JIA patients. The results showed that blueberries reduced interleukins 1 (IL1) alpha and beta, and increased IL1 receptor antagonist (IL1RA), modulating immunity and inflammatory responses. Therefore, the authors suggested that combining blueberry and etanercept can reduce the severity of JIA and should be added to JIA therapy [49]. 

### 3.4. Vitamin D

Vitamin D is a fat-soluble vitamin involved in human physiological processes such as bone mineralization, insulin, and immune regulation. Vitamin D status is governed by vitamin D receptor genotype, skin tone, clothing, environmental variations in exposure to ultraviolet B radiation, and vitamin D intake [50]. As an immune and inflammatory mediator, it may significantly influence pathogenesis and the outcome of JIA [6,51,52,53]. Vitamin D tends to suppress the body’s immune response, and when in low concentrations, it is associated with an increase in pro-inflammatory mediators and more active disease [52]. Some studies identified a lower level of 25-hydroxyvitamin D in JIA patients than in healthy children [52,54,55], but it is not well explored whether vitamin D plays a role in JIA incidence by examining dietary vitamin D intake. A recent scoping review mentioned that the relationship between vitamin D and active autoimmune diseases is correlative and not causative and suggests long-term randomized studies controlling confounding variables (sun exposure and vitamin D intake) to establish the relationship between vitamin D and disease activity [52].

A cross-sectional study showed an association between long-term methotrexate (MTX) therapy in children with JIA and a lower vitamin D status, but the causal pathways were not elucidated. However, the unfavorable effect of metotrexate therapy on 25-hydroxyvitamin D may indicate the role of this particular medication in an increased risk of vitamin D deficiency. 

A study confirmed that vitamin D deficiency was common in patients with JIA and associated with higher JIA disease activity, that the progression of oligoarthritis occurred more often in patients with vitamin D deficiency than in those with sufficiency, and that vitamin D status was inversely correlated with the risk of developing uveitis [53]. A recent study evaluated the status of serum 25(OH)D in 30 newly diagnosed JIA patients from Bangladesh compared to healthy controls. Low vitamin D status was found in 60% of JIA patients and 33% of controls, with a significantly lower mean level of serum 25(OH)D than the control group. Moreover, 25(OH)D decreased considerably as disease duration increased [56].

A study verified the effect of vitamin D supplementation at 1–2  μg/kg body weight/day in 13 children with JIA [57]. It showed a significant increase in 25(OH)D but did not change the disease activity [57]. In a recent randomized controlled trial study, the supplementation of vitamin D (2000 IU/day) for 24 weeks improved the serum concentration of 25(OH)D but did not reduce JIA activity or improve patients’ bone mineral density [58]. 

The optimal vitamin D status for children with JIA is unknown, as is if their reduced status is caused by increased utilization, reduced vitamin D status in JIA patients, the impact of vitamin D on JIA activity, or the role of genetic polymorphisms with JIA. However, considering epidemiological studies and the underlying immune-modulating mechanisms, correcting vitamin D deficiency in patients with JIA with adequate vitamin D supplementation presents potential benefits, and the optimal concentration of vitamin D and the corresponding dietary requirements for patients with JIA must be determined [50,52]. 

### 3.5. Folate

Folate supplementation tends to attenuate symptoms caused by methotrexate (MTX), the leading antirheumatic drug used in JIA management. At a lower dose, MTX acts as an anti-inflammatory agent and is a folic acid analog [59]. MTX is an inhibitor of dihydrofolate reductase (DHFR) and causes the depletion of the bioactive folate pool, inhibiting folate-dependent methylation reactions and metabolizing intracellularly through the addition of glutamate residues. These polyglutamate metabolites become inhibitors of folate-dependent enzymes [60]. In a mouse model, a study confirmed the antifolate effect of MTX [60]. Another study evaluated if folate supplementation attenuates adverse side effects and affects MTX efficacy [61]. Common adverse reactions are generally nausea, vomiting, mouth sores, loss of appetite, hair loss, malaise, elevated liver function tests, and leucopenia. These have been suggested to be related to induced folate depletion. Therefore, folate supplementation has been suggested to counteract these MTX side effects. The authors concluded that folate supplementation might counteract the most common MTX side effects in JIA patients, and there is insufficient evidence to conclude whether folate supplementation affects disease medication efficacy [61].

A pilot study evaluated the influence of MTX and folic acid supplementation in 17 JIA children with hyperhomocysteinemia compared to healthy volunteers [62]. JIA patients in the MTX treatment group were randomly assigned to folic acid supplementation (1 mg/d/p.o.) followed by placebo (8 weeks each) or vice versa. The results showed that JIA patients using MTX had significantly elevated baseline plasma total homocysteine concentrations compared to healthy controls. MTX and folate supplementation had no significant impact on total homocysteine concentration, showing that hyperhomocysteinemia in JIA children was not influenced by MTX treatment and folic acid supplementation [62].

The Methotrexate Advice and Recommendations on Juvenile Idiopathic Arthritis (MARAJIA) consensus recommends folic or folinic acid supplementation to prevent MTX side effects [63]. The recommended dose for folinic acid is about one-third of the MTX dose, at least 24 h after the weekly MTX. Folic acid is recommended 1 mg/day, skipping the day when MTX is administered, based on adult studies and limited pediatric data [63]. This low folic acid dose (1 mg/day) did not affect the MTX anti-inflammatory efficacy and attenuated the gastrointestinal and mucosal toxicity symptoms, and seems to be associated with a reduced MTX discontinuation rate [63]. Besides MARAJIA, the American College of Rheumatology Guideline for JIA treatment strongly recommends using folic or folinic acid in conjunction with MTX treatment [12].

### 3.6. Copper and Zinc

Copper and zinc play significant roles as cofactors in the normal immune system and integrity of the articular tissue [64]. They are crucial catalytic cofactors for several enzymes, structural proteins, and transcription factors. Their deficiencies are linked to gut inflammation, and a study showed their association with rheumatoid arthritis [11,64]. A study with 30 Egyptian JIA patients and 20 healthy children showed that serum levels of zinc were lower and those of copper were higher in JIA patients than in healthy controls. The authors suggested that the increase in serum copper may occur due to the rise in ceruloplasmin (an acute phase protein), and that its role in adjuvant arthritis is to neutralize free oxygen radicals to stop the process of turning chronic [64]. Lower levels of zinc need attention in JIA due to their importance to the immune system and because zinc is a structural element of alkaline phosphatase and stimulates its synthesis in osteoblasts, playing an important role in bone mineralization that might be impaired by JIA [64].

### 3.7. Calcium

Disease activity, duration, and inflammation play essential roles in the deterioration of mineral and bone metabolism in chronic rheumatologic disorders [65]. Insufficient bone mineralization during childhood is associated with greater risk of osteoporosis. Bone mineralization is a multifactorial process, but is largely linked to calcium intake. Adequate calcium intake is essential in developing strong bones, reducing the risk of osteoporosis [66]. Children with chronic health conditions face a greater risk for low bone mineral density (BMD) and osteoporosis in adulthood. A study confirmed that bone mineral density in JIA patients was lower than in the control group and that the steroid medication negatively affected growth and bones. The authors suggested that vitamin D and a calcium-rich diet could protect JIA patients against bone loss [21]. 

A study evaluated the presence and etiopathogenesis of osteopenia in 41 JIA patients and showed that osteopenia was present in 36.5% of patients [67]. Age, sex, age at onset, disease duration, and duration of methotrexate treatment were not related to osteopenia. The disease activity and nutritional status were associated with osteopenia. JIA patients with osteopenia presented lower serum calcium, magnesium, 25(OH)D, and vitamin D. Therefore, the authors concluded that calcium, magnesium, and vitamin D deficits were related to osteopenia in JIA patients [67]. 

A randomized controlled trial evaluated the effects of daily calcium supplementation with vitamin D (1000 mg of elemental Ca + 400 IU of vitamin D) on BMD of 198 non-corticosteroid-treated JIA patients for 24 months [68]. Although calcium supplementation slightly increased BMD compared with placebo in JIA children, the authors concluded that the results do not provide strong support for the routine use of calcium supplementation in JIA patients [68].

### 3.8. Iron

JIA tends to be accompanied by anemia, mainly microcytic, which may often be indistinguishable from anemia due to iron deficiency [69]. The prevalence of anemia in JIA patients ranges from 35.8% to 52.3% [70]. In JIA, anemia is different and more severe than anemia in adults with rheumatoid arthritis [71,72]. 

A study evaluated eight JIA patients with severe persistent anemia unresponsive to oral iron therapy and treated them with intravenous iron saccharate [71]. The authors showed that the anemia was mostly related to IL-6-induced iron sequestration in the reticuloendothelial system [71]. Despite the association of JIA anemia with high levels of IL-6, the pathogenesis is not totally clear. The potential mechanism of iron malabsorption observed in JIA patients occurs by IL-6 stimulating hypoxia-induced erythropoietin production and erythroid progenitor proliferation and increasing ferritin expression and hepatic uptake of serum iron, causing a reticuloendothelial iron block. In addition, IL-6 induces the liver production of peptide that inhibits iron absorption in the intestine and releases recycled iron from the macrophages [73,74].

A study evaluated 20 JIA patients with low hemoglobin levels (Hb) [75]. All the patients presented high levels of IL-6. The authors showed that Hb level was directly related to corpuscular volume and inversely related to circulating transferrin receptor, suggesting that the anemia severity was associated with the degree of iron-deficient erythropoiesis. In 10 severely anemic JIA patients, anemia was ameliorated with intravenous iron administration. The iron deficiency was caused by decreased iron absorption, complicating long-lasting inflammation in anemic patients. The authors mentioned that oral iron supplementation did not recover anemia in JIA patients. However, intravenous iron saccharate was considered safe and effective in anemia correction [75]. 

### 3.9. Probiotics

In JIA, genetics, the environment, diet, and lifestyle also affect the gut microbial profiles, potentially causing dysbiosis and gut epithelial permeability. These may induce the production of interleukin-17 (IL-17), which migrates from the gut to peripheral lymphoid tissue. IL-17 promotes germinal center B-cell differentiation in systemic organs, producing autoantibodies that may circulate into target organ joints, contributing to the development or maintenance of inflammation. Therefore, gut dysbiosis and permeability are gaining interest as potential pathogenetic factors for JIA [76]. 

It is known that probiotics can lead to immunomodulation via gut microflora alterations. Their use can change cytokine levels, favoring an anti-inflammatory response, an increase in IL-10 and transforming growth factor (TGF)-β, and a decrease in tumor necrosis factor (TNF)-α [77]. Therefore, some studies have been conducted to verify if probiotics exert beneficial effects on JIA treatment. A proof-of-concept study evaluated the effects of probiotics (composed of *Streptococcus thermophilus*, *Bifidobacterium breve*, *B. longum*, *B. infantis*, *Lactobacillus acidophilus*, *L. plantarum*, *L. paracasei*, and *L. delbrueckii)* on immune and clinical parameters of JIA patients compared to controls for 12 weeks. Serum IL-6 levels decreased in the probiotic group and serum IL-10 levels increased in the placebo group, but the probiotic use did not significantly change immune parameters compared to placebo. Therefore, in this study, the probiotic therapy in JIA patients did not show significant immune or clinical effects [77]. 

Another study evaluated microbiota and if probiotics affect fecal microbiota in enthesitis-related arthritis JIA (ERA-JIA) patients [78]. The patients received one capsule of a probiotic (composed of *Streptococcus thermophilus*, *Bifidobacterium breve*, *B. longum*, *B. infantis*, *Lactobacillus acidophilus*, *L. plantarum*, *L. paracasei*, and *L. delbrueckii*) twice daily for 12 weeks, and after the intervention, they were compared to the control group. The study showed dysbiosis in the guts of ERA-JIA patients with increased bacterial groups that may promote a pro-inflammatory state, suggesting that alteration of microbiota can change the gut and the systemic immune response, playing a role in the causation of ERA-JIA. Therefore, the authors stated that restoring gut microbiota to normal may help ameliorate ERA-JIA [78]. However, probiotic supplementation in this patient group did not significantly reverse inflammation and systemic immune response. The authors suggested further studies using a higher-than-usual probiotic dose and different formulations of a probiotic in ERA-JIA treatment. More studies are needed to search for therapies to alter gut microbiomes potentially valuable for JIA treatment since evidence for probiotics in JIA is not conclusive.

**Figure 1:**
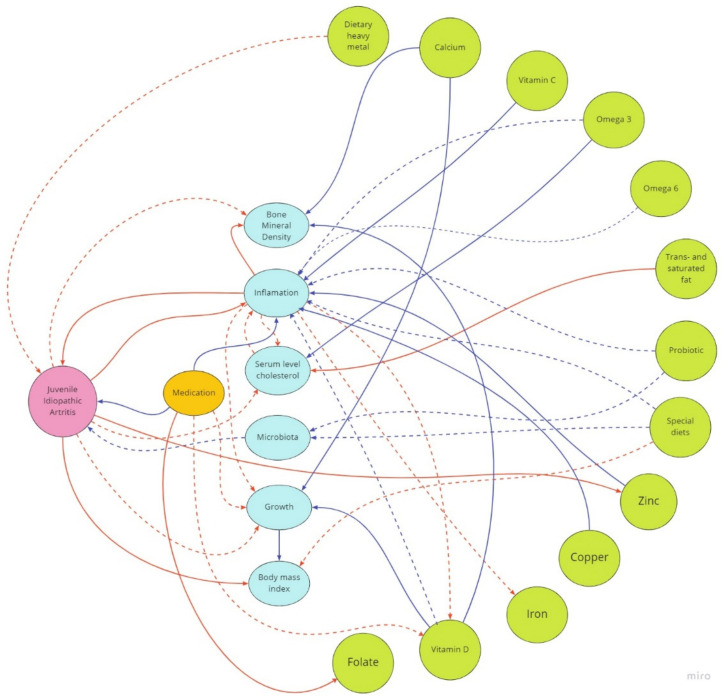
Potential effects of dietary components on JIA patients’ growth, inflammation, bone mineral density, body mass index, microbiota, and medication. Blue lines—positive effect; red lines—negative effect; dashed blue lines—potential positive effect; dashed red lines—potential negative effect.

Figure 1 summarizes the potential effects of dietary components on JIA patients’ growth, inflammation, bone mineral density, body mass index, microbiota, and medication found in this review.

It is important to emphasize that the reviewed studies generally present results regarding a specific nutrient or restrictive diet in relation to body recovery and reducing inflammation in JIA patients. Moreover, the impact of nutrition is difficult to assess and interpret in children and adolescents, particularly those with JIA, due to family influence, dietary regulation, and data collection [79]. However, the importance of a healthy and adequate diet is recognized in JIA patients’ development, growth, and health maintenance. Therefore, diet should be composed of enough macro- and micronutrients and include fruits, vegetables, and healthy fats and be limited in fat, artificial ingredients, fried foods, salt, and sugar to help JIA patients minimize the risk of additional health problems [29,31,46]. Considering that JIA is a type of rheumatoid arthritis (RA) that occurs in children and adolescents, it is speculated that the effects of nutrients help the body recover and reduce inflammation in JIA patients, as well as in adult RA patients [80]. Diet may influence susceptibility to autoimmune diseases by epigenetic mechanisms and can aggravate or protect RA patients from inflammation and symptoms [80]. Well-designed interventional studies of nutrients and dietary patterns are necessary to determine the impact of diet on improving symptoms and growth patterns in JIA patients.

## 4. Conclusions

Dietary aspects play essential roles in JIA patients’ growth, BMI, BMD, inflammation, and recovery. Studies are diverse and most analyze the effect of a single nutrient on JIA. Moreover, the impacts of diet and nutrition are difficult to interpret in the pediatric population due to family influence, dietary regulation, and data collection in children/adolescents. Suboptimal nutrition seems to adversely affect the long-term outcome of JIA patients. Nutritional deficiency potentially affects the JIA patient’s general wellbeing and disease control and contributes to growth, inflammation, BMI, and BMD disturbances. It was also possible to verify that the correct status of nutrients helps the body recover and reduces inflammation in JIA patients, since nutritional status and nutrients play an important role in regulating immune function. Therefore, despite the lack of standardization among studies, the potential benefits of a healthy diet on short- and long-term health and wellbeing in JIA patients are noteworthy.

## Data Availability

Not applicable.

## References

[1] Martini A., Lovell D.J., Albani S., Brunner H.I., Hyrich K.L., Thompson S.D., Ruperto N. (2022). Juvenile Idiopathic Arthritis. Nat. Rev. Dis. Prim..

[2] Simon T.A., Priya Harikrishnan G., Kawabata H., Singhal S., Brunner H.I., Lovell D.J. (2020). Prevalence of Co-Existing Autoimmune Disease in Juvenile Idiopathic Arthritis: A Cross-Sectional Study. Pediatr. Rheumatol..

[3] Martini A., Prakken B., Albani S. (2011). Arthritis 3 Juvenile Idiopathic Arthritis. Lancet.

[4] McCurdy D., Parsa M.F. (2021). Updates in Juvenile Idiopathic Arthritis. Adv. Pediatr..

[5] Juvenile Idiopathic Arthritis|Pediatric Orthopaedic Society of North America (POSNA). https://posna.org/Physician-Education/Study-Guide/Juvenile-Idiopathic-Arthritis.

[6] Stawicki M.K., Abramowicz P., Góralczyk A., Młyńczyk J., Kondratiuk A., Konstantynowicz J. (2022). Prevalence of Vitamin D Deficiency in Patients Treated for Juvenile Idiopathic Arthritis and Potential Role of Methotrexate: A Preliminary Study. Nutrients.

[7] Juvenile Idiopathic Arthritis (JIA)|Arthritis Foundation. https://www.arthritis.org/diseases/juvenile-idiopathic-arthritis.

[8] Pelajo C.F., Lopez-Benitez J.M., Miller L.C. (2012). Obesity and Disease Activity in Juvenile Idiopathic Arthritis. Pediatr. Rheumatol..

[9] Caetano M.C., Ortiz T.T., Terreri M.T.S.L.R.A., Sarni R.O.S., Silva S.G.L., Souza F.I.S., Hilário M.O.E. (2009). Inadequate Dietary Intake of Children and Adolescents with Juvenile Idiopathic Arthritis and Systemic Lupus Erythematosus. J. Pediatr..

[10] Lofthouse C.M., Azad F., Baildam E.M., Akobeng A.K. (2002). Measuring the Nutritional Status of Children with Juvenile Idiopathic Arthritis Using the Bioelectrical Impedance Method. Rheumatology.

[11] Cleary A.G., Lancaster G.A., Annan F., Sills J.A., Davidson J.E. (2004). Nutritional Impairment in Juvenile Idiopathic Arthritis. Rheumatology.

[12] Onel K.B., Horton D.B., Lovell D.J., Shenoi S., Cuello C.A., Angeles-Han S.T., Becker M.L., Cron R.Q., Feldman B.M., Ferguson P.J. (2022). 2021 American College of Rheumatology Guideline for the Treatment of Juvenile Idiopathic Arthritis: Therapeutic Approaches for Oligoarthritis, Temporomandibular Joint Arthritis, and Systemic Juvenile Idiopathic Arthritis. Arthritis Care Res..

[13] Little E.M.I., Grevich S., Huber J.L., Suskind D.L., Bradford M.C., Stevens A.M., Zhao Y. (2019). Parental Perception of Dietary Intervention in Juvenile Idiopathic Arthritis. J. Altern. Complement. Med..

[14] Kindgren E., Guerrero-Bosagna C., Ludvigsson J. (2019). Heavy Metals in Fish and Its Association with Autoimmunity in the Development of Juvenile Idiopathic Arthritis: A Prospective Birth Cohort Study. Pediatr. Rheumatol. Online J..

[15] Hari A., Rostom S., Hassani A., el Badri D., Bouaadi I., Barakat A., Chkirat B., Elkari K., Amine B., Hajjaj-Hassouni N. (2015). Body Composition in Children with Juvenile Idiopathic Arthritis: Effect of Dietary Intake of Macronutrient: Results from a Cross Sectional Study. Pan Afr. Med. J..

[16] Gaspari S., Marcovecchio M.L., Breda L., Chiarelli F. (2011). Growth in Juvenile Idiopathic Arthritis: The Role of Inflammation. Clin. Exp. Rheumatol..

[17] Simon D., Fernando C., Czernichow P., Prieur A.-M. (2002). Linear Growth and Final Height in Patients with Systemic Juvenile Idiopathic Arthritis Treated with Longterm Glucocorticoids. J. Rheumatol..

[18] Bacon M.C., White P.H., Raiten D.J., Craft N., Margolis S., Levander O.A., Taylor M.L., Lipnick R.N., Sami S. (1990). Nutritional Status and Growth in Juvenile Rheumatoid Arthritis. Semin. Arthritis Rheum..

[19] Guzman J., Kerr T., Ward L.M., Ma J., Oen K., Rosenberg A.M., Feldman B.M., Boire G., Houghton K., Dancey P. (2017). Growth and Weight Gain in Children with Juvenile Idiopathic Arthritis: Results from the ReACCh-Out Cohort. Pediatr. Rheumatol..

[20] McErlane F., Carrasco R., Kearsley-Fleet L., Baildam E.M., Wedderburn L.R., Foster H.E., Ioannou Y., Chieng S.E.A., Davidson J.E., Thomson W. (2018). Growth Patterns in Early Juvenile Idiopathic Arthritis: Results from the Childhood Arthritis Prospective Study (CAPS). Semin. Arthritis Rheum..

[21] Okumus O., Erguven M., Deveci M., Yilmaz O., Okumus M. (2008). Growth and Bone Mineralization in Patients with Juvenile Idiopathic Arthritis. Indian J. Pediatr..

[22] Polito C., Strano C.G., Olivieri A.N., Alessio M., Iammarrone C.S., Todisco N., Papale M.R. (1997). Growth Retardation in Non-Steroid Treated Juvenile Rheumatoid Arthritis. Scand. J. Rheumatol..

[23] Bechtold S., Simon D. (2014). Growth Abnormalities in Children and Adolescents with Juvenile Idiopathic Arthritis. Rheumatol. Int..

[24] Roth J., Bechtold S., Borte G., Dressler F., Girschick H.J., Borte M. (2007). Osteoporosis in Juvenile Idiopathic Arthritis—A Practical Approach to Diagnosis and Therapy. Eur. J. Pediatr..

[25] Bechtold S., Ripperger P., Bonfig W., Schmidt H., Bitterling H., Häfner R., Schwarz H.P. (2004). Bone Mass Development and Bone Metabolism in Juvenile Idiopathic Arthritis: Treatment with Growth Hormone for 4 Years. J. Rheumatol..

[26] Knops N., Wulffraat N., Lodder S., Houwen R., Meer K. (1999). Resting Energy Expenditure and Nutritional Status in Children with Juvenile Rheumatoid Arthritis-PubMed. J. Rheumatol..

[27] Wiȩch P., Sałacińska I., Bazaliński D., Dabrowski M. (2018). Body Composition and Phase Angle as an Indicator of Nutritional Status in Children with Juvenile Idiopathic Arthritis. Pediatr. Rheumatol. Online J..

[28] Haugen M.A., Høeraal H.M., Larsed S., Gilboe I.M., Trygg K. (2009). Nutrient Intake and Nutritional Status in Children with Juvenile Chronic Arthritis. Scand. J. Rheumatol..

[29] Merwin S., Mackey E., Sule S. (2021). US NHANES Data 2013–2016: Increased Risk of Severe Obesity in Individuals with History of Juvenile Idiopathic Arthritis. Pediatr. Rheumatol..

[30] Grönlund M.M., Kaartoaho M., Putto-Laurila A., Laitinen K. (2014). Juvenile Idiopathic Arthritis Patients with Low Inflammatory Activity Have Increased Adiposity. J. Clin. Med..

[31] Weiss J.E., Ilowite N.T. (2007). Juvenile Idiopathic Arthritis. Rheum. Dis. Clin. North Am..

[32] Consolaro A., Giancane G., Alongi A., van Dijkhuizen E.H.P., Aggarwal A., Al-Mayouf S.M., Bovis F., de Inocencio J., Demirkaya E., Flato B. (2019). Phenotypic Variability and Disparities in Treatment and Outcomes of Childhood Arthritis throughout the World: An Observational Cohort Study. Lancet Child Adolesc. Health.

[33] Haugen M.A., Kjeldsen-Kragh J., Skakkebæk N., Landaas S., Sjaastad O., Movinkel P., Førre O. (1993). The Influence of Fast and Vegetarian Diet on Parameters of Nutritional Status in Patients with Rheumatoid Arthritis. Clin. Rheumatol..

[34] Kjeldsen-Kragh J. (1999). Rheumatoid Arthritis Treated with Vegetarian Diets. Am. J. Clin. Nutr..

[35] Aalto K., Lahdenne P., Kolho K.-L. (2011). Gluten-Free Diet in Juvenile Idiopathic Arthritis. Rheumatology.

[36] Berntson L., Öman A., Engstrand L., Dicksved J. (2022). A Pilot Study Investigating Faecal Microbiota After Two Dietary Interventions in Children with Juvenile Idiopathic Arthritis. Curr. Microbiol..

[37] Berntson L. (2021). A Pilot Study of Possible Anti-Inflammatory Effects of the Specific Carbohydrate Diet in Children with Juvenile Idiopathic Arthritis. Pediatr. Rheumatol..

[38] Onel K.B., Horton D.B., Lovell D.J., Shenoi S., Cuello C.A., Angeles-Han S.T., Becker M.L., Cron R.Q., Feldman B.M., Ferguson P.J. (2022). 2021 American College of Rheumatology Guideline for the Treatment of Juvenile Idiopathic Arthritis: Recommendations for Nonpharmacologic Therapies, Medication Monitoring, Immunizations, and Imaging. Arthritis Rheumatol..

[39] Gheita T., Kamel S., Helmy N., El-Laithy N., Monir A. (2012). Omega-3 Fatty Acids in Juvenile Idiopathic Arthritis: Effect on Cytokines (IL-1 and TNF-α), Disease Activity and Response Criteria. Clin. Rheumatol..

[40] Gorczyca D., Postępski J., Czajkowska A., Paściak M., Prescha A., Olesińska E., Gruenpeter A., Lachór-Motyka I., Szponar B. (2017). The Profile of Polyunsaturated Fatty Acids in Juvenile Idiopathic Arthritis and Association with Disease Activity. Clin. Rheumatol..

[41] Marangoni R.G., Hayata A.L., Borba E.F., Azevedo P.M., Bonfá E., Goldenstein-Schainberg C. (2011). Decreased High-Density Lipoprotein Cholesterol Levels in Polyarticular Juvenile Idiopathic Arthritis. Clinics.

[42] Bohr A.H., Pedersen F.K., Nielsen C.H., Müller K.G. (2016). Lipoprotein Cholesterol Fractions Are Related to Markers of Inflammation in Children and Adolescents with Juvenile Idiopathic Arthritis: A Cross Sectional Study. Pediatr. Rheumatol..

[43] Douglas W., Rodrigues R. (2021). Biomarkers of Lipid Metabolism in Patients with Juvenile Idiopathic Arthritis: Relationship with Subtype and Inammatory Activity. Pediatr. Rheumatol..

[44] Shen C.C., Yao T.C., Yeh K.W., Huang J.L. (2013). Association of Disease Activity and Anti-Rheumatic Treatment in Juvenile Idiopathic Arthritis with Serum Lipid Profiles: A Prospective Study. Semin. Arthritis Rheum..

[45] Jednacz E., Rutkowska-Sak L. (2015). Assessment of the Body Composition and Parameters of the Cardiovascular Risk in Juvenile Idiopathic Arthritis. Biomed. Res. Int..

[46] Rondanelli M., Perdoni F., Peroni G., Caporali R., Gasparri C., Riva A., Petrangolini G., Faliva M.A., Infantino V., Naso M. (2021). Ideal Food Pyramid for Patients with Rheumatoid Arthritis: A Narrative Review. Clin. Nutr..

[47] Perkins A., Sontheimer C., Otjen J.P., Shenoi S. (2020). Scurvy Masquerading as Juvenile Idiopathic Arthritis or Vasculitis with Elevated Inflammatory Markers: A Case Series. J. Pediatr..

[48] LI Y. (2019). Advances in Immunoregulatory Effects of Dietary Nutrition on Juvenile Idiopathic Arthritis. Int. J. Pediatr..

[49] Zhong Y., Wang Y., Guo J., Chu H., Gao Y., Pang L. (2015). Blueberry Improves the Therapeutic Effect of Etanercept on Patients with Juvenile Idiopathic Arthritis: Phase III Study. Tohoku J. Exp. Med..

[50] Wu C.Y., Yang H.Y., Luo S.F., Huang J.L., Lai J.H. (2022). Vitamin D Supplementation in Patients with Juvenile Idiopathic Arthritis. Nutrients.

[51] Pelajo C.F., Lopez-Benitez J.M., Miller L.C. (2010). Vitamin D and Autoimmune Rheumatologic Disorders. Autoimmun. Rev..

[52] Finch S.L., Rosenberg A.M., Vatanparast H. (2018). Vitamin D and Juvenile Idiopathic Arthritis. Pediatr. Rheumatol..

[53] Sengler C., Zink J., Klotsche J., Niewerth M., Liedmann I., Horneff G., Kessel C., Ganser G., Thon A., Haas J.P. (2018). Vitamin D Deficiency Is Associated with Higher Disease Activity and the Risk for Uveitis in Juvenile Idiopathic Arthritis-Data from a German Inception Cohort. Arthritis Res. Ther..

[54] Zou J., Thornton C., Chambers E.S., Rosser E.C., Ciurtin C. (2021). Exploring the Evidence for an Immunomodulatory Role of Vitamin D in Juvenile and Adult Rheumatic Disease. Front. Immunol..

[55] Pelajo C.F., Lopez-Benitez J.M., Miller L.C. (2011). 25-Hydroxyvitamin D Levels and Vitamin D Deficiency in Children with Rheumatologic Disorders and Controls. J. Rheumatol..

[56] Sumi S.K., Rahman S.A., Islam M.I., Islam M.M., Talukder M.K. (2020). Vitamin D Profile in Juvenile Idiopathic Arthritis Patients in a Tertiary Care Hospital in Bangladesh. Mymensingh Med. J..

[57] Reed A., Haugen M., Pachman L.M., Langman C.B. (1991). 25-Hydroxyvitamin D Therapy in Children with Active Juvenile Rheumatoid Arthritis: Short-Term Effects on Serum Osteocalcin Levels and Bone Mineral Density. J. Pediatr..

[58] Tang T., Zhang Y., Luo C., Liu M., Xu L., Tang X. (2019). Adjunctive Vitamin D for the Treatment of Active Juvenile Idiopathic Arthritis: An Open-Label, Prospective, Randomized Controlled Trial. Exp. Ther. Med..

[59] Jacobson J.L., Pham J.T. (2018). Juvenile Idiopathic Arthritis: A Focus on Pharmacologic Management. J. Pediatr. Health Care.

[60] Singh R.K., van Haandel L., Kiptoo P., Becker M.L., Siahaan T.J., Funk R.S. (2019). Methotrexate Disposition, Anti-Folate Activity and Efficacy in the Collagen-Induced Arthritis Mouse Model. Eur. J. Pharmacol..

[61] Killeen O.G., Gardner-Medwin J.M. (2006). In Juvenile Idiopathic Arthritis, Is Folate Supplementation Effective against Methotrexate Toxicity at the Expense of Methotrexate’s Efficacy?. Arch. Dis. Child..

[62] Huemer M., Födinger M., Huemer C., Sailer-Höck M., Falger J., Rettenbacher A., Bernecker M., Artacker G., Kenzian H., Lang T. (2003). Hyperhomocysteinemia in Children with Juvenile Idiopathic Arthritis Is Not Influenced by Methotrexate Treatment and Folic Acid Supplementation: A Pilot Study. Clin. Exp. Rheumatol..

[63] Ferrara G., Mastrangelo G., Barone P., la Torre F., Martino S., Pappagallo G., Ravelli A., Taddio A., Zulian F., Cimaz R. (2018). Methotrexate in Juvenile Idiopathic Arthritis: Advice and Recommendations from the MARAJIA Expert Consensus Meeting. Pediatr. Rheumatol..

[64] Yasser S.A., Hashaad N.I., Shouzan A.M., el Nouty H.A. (2016). Measurement of Serum Trace Elements Levels in Patients with Juvenile Idiopathic Arthritis. Egypt. Rheumatol. Rehabil..

[65] Lin Z., Li W. (2016). The Roles of Vitamin D and Its Analogs in Inflammatory Diseases. Curr. Top. Med. Chem..

[66] Stark L.J., Janicke D.M., McGrath A.M., Mackner L.M., Hommel K.A., Lovell D. (2005). Prevention of Osteoporosis: A Randomized Clinical Trial to Increase Calcium Intake in Children with Juvenile Rheumatoid Arthritis. J. Pediatr. Psychol..

[67] Rusu T.E., Murgu A., Moraru E., Florea M.M., Ioniuc I., Alexoaie M., Ruginǎ A., Goţia S. (2008). [Osteopenia in Children with Juvenile Idiopathic Arthritis]. Rev. Med. Chir. Soc. Med. Nat. Iasi.

[68] Lovell D.J., Glass D., Ranz J., Kramer S., Huang B., Sierra R.I., Henderson C.J., Passo M., Graham B., Bowyer S. (2006). A Randomized Controlled Trial of Calcium Supplementation to Increase Bone Mineral Density in Children with Juvenile Rheumatoid Arthritis. Arthritis Rheum..

[69] Kivivuori S.M., Pelkonen P., Ylijoki H., Verronen P., Siimes M.A. (2000). Elevated Serum Transferrin Receptor Concentration in Children with Juvenile Chronic Arthritis as Evidence of Iron Deficiency. Rheumatology.

[70] Wilson A., Yu H.T., Goodnough L.T., Nissenson A.R. (2004). Prevalence and Outcomes of Anemia in Rheumatoid Arthritis: A Systematic Review of the Literature. Am. J. Med..

[71] Martini A., Ravelli A., di Fuccia G., Rosti V., Cazzola M., Barosi G. (1994). Intravenous Iron Therapy for Severe Anaemia in Systemic-Onset Juvenile Chronic Arthritis. Lancet.

[72] Ravelli A., Martini A. (2007). Juvenile Idiopathic Arthritis. Lancet.

[73] Martini A. (2012). Systemic Juvenile Idiopathic Arthritis. Autoimmun. Rev..

[74] Isaacs J.D., Harari O., Kobold U., Lee J.S., Bernasconi C. (2013). Effect of Tocilizumab on Haematological Markers Implicates Interleukin-6 Signalling in the Anaemia of Rheumatoid Arthritis. Arthritis Res. Ther..

[75] Cazzola M., Ponchio L., Benedetti F., Ravelli A., Rosti V., Beguin Y., Invernizzi R., Barosi G., Martini A. (1996). Defective Iron Supply for Erythropoiesis and Adequate Endogenous Erythropoietin Production in the Anemia Associated with Systemic-Onset Juvenile Chronic Arthritis. Blood.

[76] De Filippo C., di Paola M., Giani T., Tirelli F., Cimaz R. (2019). Gut Microbiota in Children and Altered Profiles in Juvenile Idiopathic Arthritis. J. Autoimmun..

[77] Shukla A., Gaur P., Aggarwal A. (2016). Effect of Probiotics on Clinical and Immune Parameters in Enthesitis-Related Arthritis Category of Juvenile Idiopathic Arthritis. Clin. Exp. Immunol..

[78] Aggarwal A., Sarangi A.N., Gaur P., Shukla A., Aggarwal R. (2017). Gut Microbiome in Children with Enthesitis-Related Arthritis in a Developing Country and the Effect of Probiotic Administration. Clin. Exp. Immunol..

[79] Zare N., Mansoubi M., Coe S., Naja A.A., Bailey K., Harrison K., Sheehan J., Dawes H., Barker K. (2022). An Investigation into the Relationship between Dietary Intake, Symptoms and Health-Related Quality of Life in Children and Young People with Juvenile Idiopathic Arthritis: A Systematic Review and Meta-Analysis. Res. Square.

[80] Conigliaro P., Triggianese P., de Martino E., Fonti G.L., Chimenti M.S., Sunzini F., Viola A., Canofari C., Perricone R. (2019). Challenges in the Treatment of Rheumatoid Arthritis. Autoimmun. Rev..

